# Gene Expression Profile of Endotoxin-stimulated Leukocytes of the Term New Born: Control of Cytokine Gene Expression by Interleukin-10

**DOI:** 10.1371/journal.pone.0053641

**Published:** 2013-01-11

**Authors:** Dennis Davidson, Alla Zaytseva, Veronika Miskolci, Susana Castro-Alcaraz, Ivana Vancurova, Hardik Patel

**Affiliations:** 1 Division of Neonatal-Perinatal Medicine, Stony Brook Long Island Children's Hospital, Stony Brook, New York, United States of America; 2 The Feinstein Institute for Medical Research, Manhasset, New York, United States of America; 3 Department of Biology, St John's University, New York, New York, United States of America; University of North Dakota, United States of America

## Abstract

**Introduction:**

Increasing evidence now supports the association between the fetal inflammatory response syndrome (FIRS) with the pathogenesis of preterm labor, intraventricular hemorrhage and bronchopulmonary dysplasia. Polymorphonuclear leukocyte (PMNs) and mononuclear cell (MONOs) infiltration of the placenta is associated with these disorders. The aim of this study was to reveal cell-specific differences in gene expression and cytokine release in response to endotoxin that would elucidate inflammatory control mechanisms in the newly born.

**Methods:**

PMNs and MONOs were separately isolated from the same cord blood sample. A genome-wide microarray screened for gene expression and related pathways at 4 h of LPS stimulation (n = 5). RT-qPCR and ELISA were performed for selected cytokines at 4 h and 18 h of LPS stimulation.

**Results:**

Compared to PMNs, MONOs had a greater diversity and more robust gene expression that included pro-inflammatory (PI) cytokines, chemokines and growth factors at 4 h. Only MONOs had genes changing expression (all up regulated including interleukin-10) that were clustered in the JAK/STAT pathway. Pre-incubation with IL-10 antibody, for LPS-stimulated MONOs, led to up regulated PI and IL-10 gene expression and release of PI cytokines after 4 h.

**Discussion:**

The present study suggests a dominant role of MONO gene expression in control of the fetal inflammatory response syndrome at 4 hrs of LPS stimulation. LPS-stimulated MONOs but not PMNs of the newborn have the ability to inhibit PI cytokine gene expression by latent IL-10 release.

## Introduction

Increasing evidence now supports the association between the fetal inflammatory response syndrome (FIRS) with the pathogenesis of preterm labor, intraventricular hemorrhage and bronchopulmonary dysplasia (BPD) [1]–[3]. Endotoxin (LPS) is one important stimulus for the FIRS and has been measured in the amniotic fluid when there is premature rupture of membranes with or without labor [4]. The pathogenesis of FIRS involves inflammation by innate immune cells, principally polymorphonuclear leukocytes (PMNs) and monocytes (MONOs) [1], [5]. PMNs and MONOs are sequentially recruited into the lung of the newborn in the early development of BPD [6]–[8]. In experimental models of premature lung disease, intra-amniotic administration of LPS accelerates lung surfactant but causes a persistent lung inflammation as seen in BPD [9]. After birth, an imbalance between airspace pro-inflammatory and anti-inflammatory mediators, particularly cytokines, is believed to be one of the principal causes for persistent inflammation in BPD [10]–[12]. Bronchopulmonary dysplasia is one of the major causes of mortality and morbidity in neonatal period.

Endogenous interleukin-10 is a potent inhibitor of pro-inflammatory cytokine release [13] that is deficient in the preterm placenta [14] as well as the preterm and term lung during the postnatal development of BPD [15]–[17]. Exogenous IL-10 has been shown to be an effective anti-inflammatory agent in adult disorders such as psoriasis and inflammatory bowel disease [18]. Experimental evidence at the cellular level suggests that exogenous IL-10 may have therapeutic efficacy in perinatal inflammatory disorders of the newborn such as BPD [16], [19].

The first aim of the present study was to compare the genome–wide gene expression of LPS on PMNs and MONOs of the newly born. The hypothesis was that cell-specific differences in the gene expression and cytokine release in response to endotoxin would reveal inflammatory control mechanisms in the new born. Subsequently, our aim focused on the role of endogenous IL-10 in the control of gene expression and pro-inflammatory cytokine release, particularly IL-6, by LPS-stimulated PMNs and MONOs of the newly born. IL-6 is a pro-inflammatory cytokine that is used as one of the principal plasma markers in cord blood to help define the FIRS [20].

## Methods

### Subjects and sample collection

Cord blood was obtained from placentas immediately after elective, term, cesarean section deliveries. Deliveries were not associated with labor, rupture of membranes, clinical chorioamnionitis, antenatal steroids, maternal disorders or maternal medications for underlying diseases. The study was approved by the Internal Review Board of the North Shore-Long Island Jewish Health System; consent was not required for discarded placentas and the data were analyzed anonymously.

### Cell isolation

PMNs and MONOs were isolated from the same cord blood sample as described previously [21], [22]. PMN purity was >95% by differential staining and light microscopy, viability was >95% by trypan blue exclusion. MONO purity was >90% as determined by flow cytometry for CD14+ cells and viability was >95% by trypan blue exclusion [21], [22].

### Cell culture

For microarray experiments, PMNs and MONOs (5×10^6^ cells) were separately suspended in RPMI 1640+10% FCS and stimulated with a clinically relevant dose of LPS from E.coli 0111:B4 (10 ng/mL) [4] (Sigma-Aldrich Corp. St. Louis, MO, USA) for 4 h at 37°C and 5% CO_2_. After 4 h cells were preserved in RNA*later* (Invitrogen, Grand Island, NY, USA) to preserve RNA. We chose 4 h based on previous work demonstrating that both pro-inflammatory and anti-inflammatory cytokines gene expressions were both up regulated at this time point [19]. For RT-qPCR and ELISA experiments, (n = 6) MONOs (2×10^6^ cells) were pre-incubated with PBS or anti IL-10 antibody (10 µg/ml, R&D systems, Minneapolis, MN, USA) or IgG antibody (10 µg/ml, R&D systems, Minneapolis, MN, USA) [21] for 1 h and then stimulated with LPS for 4 and 18 h. PBS was used as the vehicle for LPS, IgG and IL-10 antibody.

### RNA isolation, amplification and labeling

Total RNA was isolated using the Qiagen RNeasy mini kit (Qiagen, Valencia, CA, USA). Total RNA was amplified and labeled (cRNA) using the Ambion MessageAmp™ II-Biotin enhanced kit (Invitrogen, Grand Island, NY, USA). Both total RNA and cRNA concentration and quality were determined using NanoDrop-1000 (Thermo scientific, Wilmington, DE, USA) and an Agilent 2100 Bio-analyzer (Agilent Technologies, Palo Alto, CA, USA) respectively.

### Microarray hybridization and scanning

20 µg fragmented, biotin-labeled cRNA (n = 5) was hybridized to Gene Chip Human U133 plus 2.0 microarray platforms (Affymetrix, Santa Clara, CA, USA) for 18 h at 45°C and were scanned by a Gene Chip 3000 scanner (Affymetrix, Santa Clara, CA, USA).

### Microarray data analysis

Raw data (n = 5 subjects) was uploaded onto GeneSifter (Geospiza, Inc. Seattle, WA, USA), log transformed and normalized using GC-RMA. Paired t-tests (significance = p<0.05) were performed for gene expression between PBS and LPS with a Benjamini & Hochberg correction method to control the false discovery rate. Then, gene expression change was defined as a fold change of ≥1.5. KEGG (Kyoto Encyclopedia of Genes and Genomes) gene pathways (significance = z score of 2<Z<−2) were used to functionally categorize genes before further analysis of specific gene changes. Individual gene changes in both cell types were also examined based on previous work measuring cytokines and chemokines detected in cord blood of the newly born with FIRS, as well as those detected in airway fluid for neonates with evolving BPD [10], [20], [23].

### Quantitative reverse transcriptase PCR (RT-qPCR)

RT-qPCR was used to validate microarray findings and the role of IL-10 on gene expression for interleukin-6 (IL-6), tumor necrosis factor (TNF), interleukin-8 (IL-8) and integrin β8 (ITGB8) as well as IL-10 in subsequent experiments. Primers and hybridization probes were designed by Roche Universal Probe Library Assay Design Center (Roche, Mannheim, Germany) for selected genes (IL-10 primers cataaattagaggtctccaaaatcg and aaggggctgggtcagctat, UPL probe #45; IL-6 primers gatgagtacaaaagtcctgatcca and ctgcagccactggttctgt, UPL probe #40; IL-8 primers agacagcagagcacacaagc and atggttccttccggtggt, UPL probe #72; TNF primers cagcctcttctccttcctgat and gccagagggctgattagaga, UPL probe #29; ITGB8 primers gcattatgtcgaccaaacttca and gcaacccaatcaagaatgtaact, UPL probe #19). RT-qPCR was carried out using Lightcycler 480 RNA Master Hydrolysis Probes reaction mix (Roche, Mannheim, Germany) and Roche Lightcycler 480 thermocycler (Roche, Mannheim, Germany). Results were analyzed by the relative quantification method on Lightcycler 480 software 1.5. mRNA fold changes were calculated using monocytes at 0 h without any treatment. A paired t test (n = 6) was used to compare data from LPS alone versus LPS with IL-10 antibody in MONOs.

### ELISA

Interleukin-6 release (n = 6 subjects, Human IL-6 Quantikine ELISA Kit R&D systems, Minneapolis, MN, USA) was measured at 4 and 18 h in cell culture supernatant from same subjects used for RT-qPCR. A paired t test was used to compare data from LPS alone versus LPS with IL-10 antibody in MONO cell culture media.

## Results

Gene expression changes in PMNs and MONOs from 5 newborns were detected by the genome-wide microarray after 4 h of LPS stimulation. Changes in gene expression were defined as at least a 1.5 fold, statistically significant difference from the PBS control. Table 1 provides an overview of the number of gene expression changes separately and in common between PMNs and MONOs. More genes were down regulated than up regulated in both PMNs and MONOs. In addition, twice as many genes specific to MONOs had up regulated expression compared to the number of gene specific to PMNs.

**Table 1:** Numbers of gene changing expression measured by genome-wide microarray analysis after 4 hr of endotoxin stimulation in polymorphonuclear leukocytes (PMNs) and monocytes (MONOs) of the newly born (n = 5).

Gene expression	PMNs	Common	MONOs
**Up regulated**	789	608	1656
**Down regulated**	2107	1248	2562

Table 2 shows the changes in gene expression under the present experimental conditions by microarray, for inflammatory mediators that have been commonly studied in cord blood and airway fluid, in the newly born exposed to the FIRS [20] and or neonates developing BPD respectively [10], [23]. The greatest up regulated fold changes in pro-inflammatory gene expression for both cell types at 4 h of LPS stimulation was IL-6 followed by IL-1α. Gene expression for the anti-inflammatory cytokine, IL-1 receptor antagonist, was greatly elevated by fold change in PMNs and MONOs. Notably, IL-10 gene expression did not significantly increase in PMNs but was markedly increased in MONOs.

**Table 2:** Gene expression measured by genome-wide microarray profiling after 4 h of endotoxin stimulation in polymorphonuclear cells (PMNs) and monocytes (MONOs) from the newly born (N = 5).

Gene Name*	Gene ID	Gene Identifier	PMNs			MONOs		
			Fold Change		p value	Fold Change		p value
Chemokine (C-C motif) ligand 2	CCL2	S69738 g	Up	6.2	0.0154	Up	22.4	0.0010
Chemokine (C-C motif) ligand 3	CCL3	NM_002983 g	Up	3.6	0.0071	Up	3.5	0.0017
Chemokine (C-C motif) ligand 4	CCL4	NM_002984 g	Up	4.1	0.0041	Up	3.7	0.0092
Chemokine (C-C motif) ligand 5	CCL5	NM_002985 g	Up	1.7	0.0072	Up	7.2	0.0011
Chemokine (C-C motif) ligand 7	CCL7	NM_006273 g	–	2.0	NS	Up	63.5	0.0009
chemokine (C-C motif) ligand 8	CCL8	AI984980 g	–	1.2	NS	Up	63.9	<0.0001
Chemokine (C-X-C motif) ligand 10	CXCL10	NM_001565 g	–	1.1	NS	Up	26.0	0.0002
Colony stimulating factor 2 (granulocyte-macrophage)	CSF2	M11734 g	–	2.3	NS	Up	2.2	0.0092
Colony stimulating factor 3 (granulocyte)	CSF3	NM_000759 g	Up	5.6	0.0028	Up	26.9	0.0001
Integrin, beta 8	ITGB8	NM_002214 g	Up	18.13	0.0004	Up	55.3	<0.0001
Intercellular adhesion molecule 1	ICAM1	NM_000201 g	Up	3.3	0.0008	Up	3.2	0.0048
Interleukin 1 receptor antagonist	IL1RN	AW083357 g	Up	9.9	0.0122	Up	24.1	0.0013
Interleukin 1, alpha	IL1A	M15329 g	Up	29.3	0.0113	Up	10.0	0.0044
Interleukin 1, beta	IL1B	NM_000576 g	Up	6.2	0.0129	Up	2.6	0.0071
Interleukin 10	IL10	NM_000572 g	–	2.5	NS	Up	55.4	<0.0001
Interleukin 12B	IL12B	NM_002187 g	Up	3.5	0.0378	Up	16.6	0.0095
Interleukin 16 (lymphocyte chemoattractant factor)	IL16	NM_004513 g	Down	2.1	0.0363	–	1.5	NS
Interleukin 18 (interferon-gamma-inducing factor)	IL18	NM_001562 g	Down	2.3	0.0001	–	1.6	0.045
Interleukin 2 receptor, alpha	IL2RA	NM_000417 g	–	1.7	NS	Up	37.0	0.0002
Interleukin 6 (interferon, beta 2)	IL6	NM_000600 g	Up	181.8	0.0007	Up	95.7	0.0014
Interleukin 7	IL7	NM_000880 g	–	1.1	NS	Up	6.1	0.0002
Interleukin 8	IL8	NM_000584 g	–	1.2	NS	–	1.6	NS
Transforming growth factor, beta 1	TGFB1	BC000125 g	Down	1.7	0.041	Down	1.6	0.0032
Tumor necrosis factor (TNF superfamily, member 2)	TNF	NM_000594 g	Up	7.1	0.0122	Up	4.0	0.02343

* Genes were selected based on previous clinical studies of the fetal inflammatory response syndrome and bronchopulmonary dysplasia [10], [20], [23].

Table 3 shows the KEGG pathways unique or common to both cell types in which significant changes in gene expression, both up regulated and down regulated, are occurring under the present experimental conditions. The JAK/STAT signaling pathway was the only pathway in which genes changed expression in one cell type, that is MONOs, and this was only up regulation. Pathways for MAPK signaling, RIG-1-like receptor signaling, and Toll-like receptor signaling pathways had genes changing in common for both cell types but not unique to either cell type. There were no genes changing in common for both cell types for protein processing in endoplasmic reticulum and the phagosome pathways. The ubiquitin mediated proteolysis pathway was only down regulated in PMNs alone, The largest group of genes changing expression were clustered in the metabolic pathway category. For both cell types there were more than twice the number of genes down regulated compared to upregulated and MONOs had more than twice the number of genes changing expression compared to PMNs for the metabolic pathway category.

**Table 3:** Pathways for genes changing expression (fold changes) measured by genome-wide microarray after 4 h of endotoxin stimulation in polymorphonuclear leukocytes (PMNs) and monocytes (MONOs) of the newly born (N = 5).

	Unique to PMNs		Common		Unique to MONOs	
KEGG Pathway*	Up	Down	Up	Down	Up	Down
JAK/STAT signaling pathway	–	–	–	–	**24** (3.7)	–
Cytokine-cytokine receptor interaction	–	**13** (−2.0)	**22** (5.3)	**6** (−2.6)	**42** (5.0)	**16** (−2.4)
NOD-like receptor signaling pathway	**8** (4.6)	–	**11** (7.1)	–	**9** (2.0)	–
Osteoclast differentiation	**11** (3.7)	–	**11** (4.0)	–	**18** (2.8)	**25** (3.6)
Apoptosis	–	–	**10** (4.7)	–	–	–
RIG-I-like receptor signaling pathway	**6** (2.6)	–	**8** (4.2)	–	–	–
Toll-like receptor signaling pathway	–	–	**13** (6.0)	–	–	–
MAPK signaling pathway	–	–	**18** (3.8)	–	–	–
Chemokine signaling pathway	–	–	**14** (3.9)	–	**26** (3.3)	–
Fc gamma R-mediated phagocytosis	–	–	–	–	**18** (4.4)	**15** (2.1)
Metabolic pathways	–	–	–	**88** (3.6)	**64** (−2.5)	**153** (4.7)
Pentose phosphate pathway	–	–	–	**6** (3.8)	–	**6** (2.2)
Phosphatidylinositol signaling system	–	–	–	**12** (3.6)	–	**14** (2.2)
Phagosome	**12** (3.6)	–	–	–	–	**23** (2.17)
Protein processing in endoplasmic reticulum	–	–	–	–	**25** (3.7)	–
Ubiquitin-mediated proteolysis	–	**25** (4.6)	–	–	–	–

* KEGG = Kyoto Encyclopedia of Genes and Genomes. (values) are z scores with significance 2<Z<−2.

Table 4 shows individual genes that are changing expression in the JAK/STAT pathway for MONOs. All genes were significantly up regulated in this pathway. The greatest fold change involved the up regulation of IL-10, suppressor of cytokine signaling 1, leptin and interleukin-7 receptor. Multiple family members of signal transducer and activator of transcription (STAT) and suppressor of cytokine signaling (SOCS) gene expression were differentially up regulated.

**Table 4:** Gene expression, all significantly up regulated, as detected by genome-wide microarrays for the JAK/STAT signaling pathway after 4 h of endotoxin stimulation in monocytes of the newly born (N = 5).

Gene Name	Gene ID	Gene Identifier	Fold Change
Colony stimulating factor 2 (granulocyte-macrophage)	CSF2	M11734	2.2
Cytokine receptor-like factor 2	CRLF2	NM_022148	14.1
Interferon regulatory factor 9	IRF9	NM_006084	3.0
Interleukin 10	IL10	NM_000572	55.4
Interleukin 15	IL15	NM_000585	6.5
Interleukin 15 receptor, alpha	IL15RA	NM_002189	24.0
Interleukin 19	IL19	NM_013371	22.8
Interleukin 2 receptor, alpha	IL2RA	NM_000417	37.0
Interleukin 23, alpha subunit p19	IL23A	NM_016584	8.3
Interleukin 4 receptor	IL4R	NM_000418	2.6
Interleukin 7	IL7	NM_000880	6.1
Interleukin 7 receptor	IL7R	NM_002185	56.6
Signal transducer and activator of transcription 1, 91 kDa	STAT1	NM_007315	5.4
Signal transducer and activator of transcription 2, 113 kDa	STAT2	NM_005419	3.3
Signal transducing adaptor molecule (SH3 domain and ITAM motif) 2	STAM2	AI571996	2.0
Suppressor of cytokine signaling 1	SOCS1	AI056051	54.5
Suppressor of cytokine signaling 2	SOCS2	NM_003877	3.9
Suppressor of cytokine signaling 3	SOCS3	BG035761	6.0

**Figure 1:**
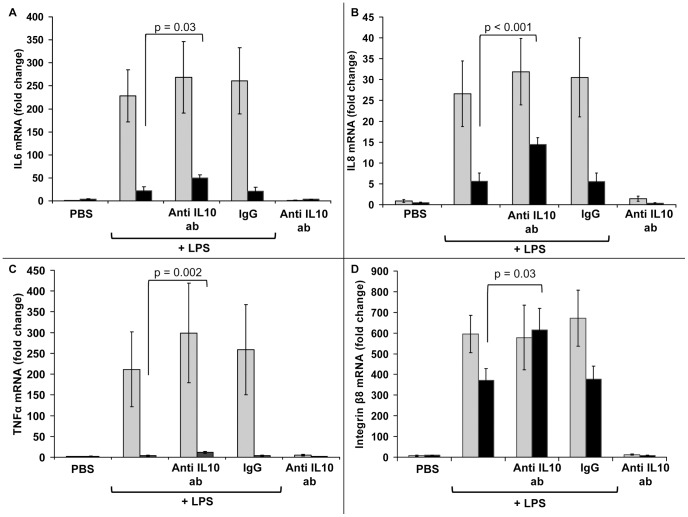
Analysis of IL-6, IL-8, TNFα and integrin β 8 mRNA by RT-qPCR in monocytes. Monocytes from the newly born were preincubated with anti-IL-10 antibody for 1 hr followed by endotoxin (LPS) stimulation for 4 h (gray bars) and 18 h (black bars). PBS alone, anti-IL-10 antibody (ab) without LPS stimulation and IgG with LPS served as controls. Values are mean ± SE (n = 6).

**Figure 2:**
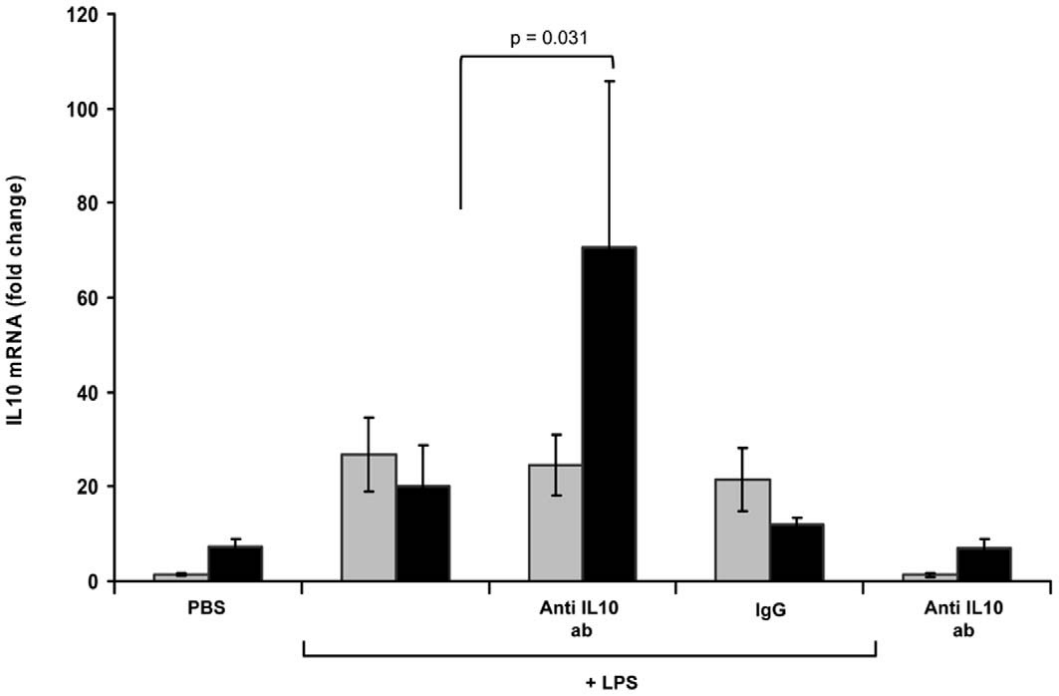
Fold changes in gene expression of IL-10 in monocytes measured by RT-qPCR. Monocytes from the newly born were preincubated with anti-IL-10 antibody for 1 hr followed by endotoxin (LPS) stimulation for 4 h (gray bars) and 18 h (black bars). PBS alone, anti-IL-10 antibody without LPS stimulation and IgG with LPS served as controls. Values are mean ± SE (n = 6).

Figure 1 shows the effect of an IL-10 monoclonal antibody on the gene expression of 4 pro-inflammatory mediators from LPS-stimulated MONOs. Gene expression was measured by RT-qPCR. Up regulation of each mediator occurred with LPS compared to PBS. The up regulation of gene expression with exposure to the IL-10 antibody, is seen at 18 h rather than at 4 h. PBS, IL-10 antibody alone and IgG plus LPS studies served negative controls indicating specificity from the IL-10 antibody on gene expression. It was noted that the interleukin-8 gene expression did not increase significantly based on microarray results (Table 1) in response to LPS at 4 h but did increase in the these separate time course experiments with measurement by RT-qPCR. Figure 2 shows similar results to Figure 1, in LPS-stimulated MONOs, when IL-10 gene expression was measured by RT-qPCR at 4 and 18 h with and without exposure to the IL-10 antibody. IL-10 expression did not increase at 4 hours but did increase at 18 h with exposure to the IL-10 antibody.

**Figure 3:**
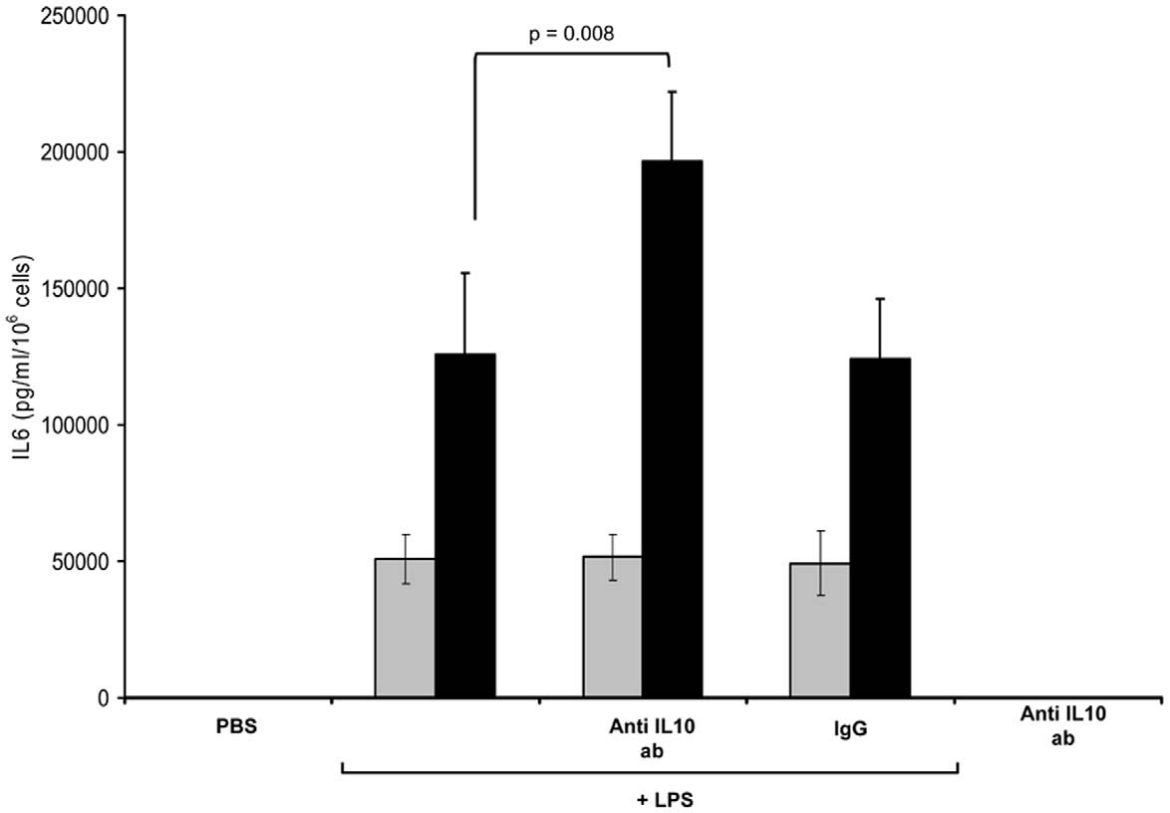
IL-6 release from monocytes of the newly born, measured by ELISA. Monocytes were preincubated with anti-IL-10 antibody for 1 hr followed by endotoxin (LPS) stimulation for 4 h (gray bars) and 18 h (black bars). PBS alone, anti-IL-10 antibody without LPS stimulation and IgG with LPS served as controls. Values are mean ± SE (n = 6).

Figure 3 shows the effect of IL-10 antibody on the release of IL-6 from LPS-stimulated MONOs with exposure to IL-10 antibody. The results are parallel to the gene expression pattern seen in Figure 2; it was only at 18 h that IL-6 levels rose with LPS and IL-10 antibody, compared to LPS alone.

## Discussion

PMNs and MONOs play a pivotal role in development of FIRS and BPD as well as preterm delivery and intraventricular hemorrhage [5], [20]. The present report provides new information that compares genome-wide profiles of LPS-stimulated PMNs and MONOs with the overall aim to better understand mechanisms of control of inflammation for the newly born. Microarray technology, used as a screening tool, demonstrated that MONOs had many more genes changing expression than PMNs upon a 4 h exposure to LPS. IL-6, one of the principal clinical marker for FIRS, had the greatest degree of upregulation in gene expression of any cytokine in both PMNs and MONOs. LPS-stimulation was associated with up regulation of JAK/STAT signaling pathway genes in MONOs but not in PMNs. Within the JAK/STAT pathway, IL-10 gene expression was significantly upregulated 55 fold in MONOs. IL-10 gene expression was not detected in PMNs. Using RT-qPCR and ELISA, it was shown that pre-incubation with IL-10 antibody before endotoxin stimulation, resulted in a up regulation of pro-inflammatory gene expression and pro-inflammatory mediator release as well as gene expression of IL-10 itself in MONOs. These effects by IL-10 were not seen at 4 h but rather 18 h of LPS stimulation.

The experimental design of this study took into account clinically and technically relevant considerations. LPS is believed to be one of the most important stimuli for FIRS, since it has been measured in the amniotic fluid of mothers with preterm rupture of membranes with and without labor in the range of 0.6 to 48 ng/ml [4]. Organisms such as mycoplasma and anaerobes could produce a different gene expression pattern. In the present study, PMNs and MONOs were exposed to LPS at 10 ng/ml, a level reported near the median of measurements taken in amniotic fluid [4]. The m-RNA transcriptome of FIRS has been described for umbilical cord blood of preterm infants with FIRS [20]; similarities to endotoxin models and pediatric sepsis were concluded but specific cell sources for the results were not studied. PMNs and MONOs are considered the two principal circulating cell types that are critical to the early development of the FIRS [5]. PMNs and MONOs are also the cells which are sequentially recruited into the lung during the development of BPD [6], [7], [9]. In preclinical studies intra-amniotic LPS can produce a persistent inflammatory airway process in the newborn [9]. The microarrays for PMNs and MONOs used in the present study included the entire genome for the detection of gene expression. Five microarrays for each cell type and condition (PBS *vs* LPS) were employed to get a high level of result specificity [24]. Unlike studies using peripheral blood monocytic cells (which contain more lymphocytes than monocytes), the present study used an isolation technique that gave >90% monocyte purity.

A limitation of this study was that only 1 time point (4 h) was analyzed with the genome-wide microarray. This time point was based on preliminary work indicating a time when pro-inflammatory followed by anti-inflammatory genes were both likely to have been expressed [19] with LPS-stimulation. Although there are many functional similarities between leukocytes of the preterm and term infant, cord blood from healthy term infants, as opposed to preterm infants could be a considered a limitation of this study. However, toll-like receptor stimulation of PMNs, isolated from pre-term and term infant samples, induce similar level of elevated interleukin 8 release compared to PMNs from adults [25]. Cells from term infants were also employed to avoid the effects of antenatal steroids, as well as maternal disorders and medications which could confound interpretation of physiologic mechanisms of innate inflammation and its control.

Three previous FIRS and BPD clinical studies [10], [20], [23] were used to list up regulated gene expression that had clinical relevance as markers to these disorders, with our *in vitro* study. IL-6 is one of the principal markers for FIRS [20] and is found early in the airway fluid of newborns who will develop BPD [26]. In the present study, IL-6 followed by interleukin-1α had the greatest fold changes for interleukin gene expression that is up regulation, by both LPS-stimulated PMNs and MONOs. However, LPS-stimulated MONOs had almost twice as many significant up regulated genes changes compared to PMNs, in common with this clinically relevant list of disease markers, which includes chemokines, growth factors, adhesion molecules and interleukins. Two anti-inflammatory mediators IL-10 and IL-1 receptor antagonist were on the list but in the present study IL-10 gene expression was only observed in LPS-stimulated MONOs, whereas both cell types expressed IL-1 receptor antagonist.

KEGG pathway grouping of the microarray gene profiling revealed that for the JAK/STAT pathway, only MONOs exhibited gene expression changes, all up regulation. The JAK/STAT pathway is a principal signaling path for many growth factors and cytokines. The JAK family of tyrosine kinase, activate STATs which transolcate from the cytoplasm to the nucleus and serve to modulate transcription [21], [27]. Exogenous IL-10 induces the translocation of phosphorylated STAT3 along with activation of the transcription factor activator protein 1 in LPS-stimulated MONOs of the newly born [21]. The anti-inflammatory action of IL-10 requires the STAT pathway [27]. Interestingly, suppressor of cytokine signaling 1 (SOCS1) was also greatly up regulated in LPS-stimulated MONOs in the present study. SOCS1 is a negative regulator of cytokine signaling particularly inhibiting interferon gamma activity [27].

To determine the effect of endogenous IL-10 release from MONOs on their robust the gene expression and release of pro-inflammatory cytokines compared to PMNs, we pretreated MONOs with a monoclonal IL-10 antibody before stimulation with LPS. The results of these studies indicated that endogenous IL-10 production and release leads to a decrease in pro-inflammatory gene expression for IL-1β, IL-8, IL-6 and TNF. In addition, endogenous IL-10 production and release decreases its own gene expression. These effects of IL-10 antibody were not observed at 4 hours but rather at 18 hours after LPS-stimulation of MONOs indicating that IL-10 is release occurs later than certain PI cytokines [21]. The effects of IL-10 antibody on gene expression were reflected in an increase in IL-6 protein release from MONOs at 18 h but not 4 h.

The temporal relationship between the initiation, adaptation, and resolution of inflammation involves changes bioenergetics [28], accordingly it was of interest that the KEGG metabolic pathway demonstrated the largest number of genes changing expression in PMNs and this occurred to even a greater extent in MONOs. Within the metabolic pathways, changes in gene expression for PMNs involved down regulation of genes related to the citrate cycle and oxidative phosphorylation. Metabolic pathways for MONOs involved down regulation of genes related to glycolysis, pentose phosphate pathway, citrate cycle, and oxidative phosphorylation. Reprogramming of the metabolic pathways, in the temporal changes of the innate immune response has been described as cellular “hibernation” [28].

In conclusion, the present study indicated that PMNs of the newly born, the first innate immune cell type invading the fetal membranes, placenta, and umbilical cord [29], as well as the airway of the newborn with evolving BPD [6], [7], [9], do not have the ability to control inflammation by an IL-10 mechanism after LPS stimulation. However, LPS-stimulated MONOs of the newly born, recruited after PMNs into these tissues, do have a late onset ability to control inflammation by an IL-10 mechanism, associated with up regulation of gene expression in the JAK/STAT pathway. This conclusion is important in the context of studies which have demonstrated absent or very low levels of IL-10 in preterm and term infants with evolving BPD [15]–[17]. Furthermore, MONOs are the precursors for alveolar macrophages as well as dendritic, microglial, Langerhans and Kupffer cells. Further studies may find that enhancement of endogenous IL-10 release or use of exogenous IL-10 could have therapeutic potential for serious inflammatory disorders [30] in the perinatal period such as preterm labor [31], or white matter injury and BPD in the newborn.
